# Tracking the Migratory Life History of Brown Croaker (*Miichthys miiuy*) Through Otolith Microchemistry in the East China Sea

**DOI:** 10.3390/ani15213129

**Published:** 2025-10-29

**Authors:** Jiarong Shen, Zeyu Xiao, Rijin Jiang, Zhongya Xuan, Yongdong Zhou, Wenjia Li, Haoran Wang, Jian Yang, Mingyuan Cui

**Affiliations:** 1 Zhejiang Marine Fisheries Research Institute, Zhoushan Field Comprehensive Scientific Observation and Research Station of the Ministry of Agriculture and Rural Affairs, Key Laboratory of Sustainable Utilization of Technology Research for Fishery Resources of Zhejiang Province, Zhoushan 316021, China; sdrzshenjiarong@163.com (J.S.); xiaozeyu327@163.com (Z.X.); zyd511@126.com (Y.Z.); lecia_wj@163.com (W.L.); 15231725895@163.com (H.W.); 17864202002@163.com (M.C.); 2 Marine and Fisheries Research Institute, Zhejiang Ocean University, Zhoushan 316021, China; 3 Key Laboratory of Freshwater Fisheries and Germplasm Resources Utilization, Ministry of Agriculture and Rural Affairs, Freshwater Fisheries Research Center, Chinese Academy of Fishery Sciences, Wuxi 214081, China; 2017213002@njau.edu.cn (Z.X.); jiany@ffrc.cn (J.Y.)

**Keywords:** *Miichthys miiuy*, migratory life history, otolith chemistry, natal sources, East China Sea

## Abstract

**Simple Summary:**

Brown croaker (*Miichthys miiuy*) is an important fishery species in the East China Sea, yet little is known about its natal origin and migration pathways. In this study, we analyzed chemical signatures in otoliths to determine natal origins and habitat use throughout the life cycle. We identified four distinct life history strategies, indicating that some individuals remain in coastal waters while others migrate among estuarine, coastal, and offshore environments. These findings reveal that *M. miiuy* exhibits high ecological flexibility and strong habitat connectivity. Such information is essential for protecting critical spawning and nursery areas and for developing more effective seasonal management strategies to support sustainable fisheries.

**Abstract:**

Brown croaker (*Miichthys miiuy*) is an economically, ecologically, and culturally important species in the East China Sea (ECS); however, populations of *M. miiuy* have declined in recent years due to climate change and high fishing intensity. Our limited understanding of wild *M. miiuy*’s migratory life history hampers effective population conservation. To meet this need, and to elucidate the migratory life history of wild *M. miiuy*, we quantified the elemental composition of otolith samples using laser ablation inductively coupled plasma mass spectrometry (LA-ICP-MS). This approach, combined with analysis of otolith microstructure, was used to evaluate the feasibility of using the Mg:Ca of otoliths chemical clock for *M. miiuy*. Using cluster analysis alongside bivariate time series analysis, we identified natal sources and reconstructed migratory histories. The results showed that consistent, periodic fluctuation of Mg:Ca ratios in otolith profiles can be used as a chemical index to indicate the age and life history stage of *M. miiuy*. Natal sources of *M. miiuy* originated from three distinct water environments: estuary (14.2%), coastal mixed waters (57.3%), and coastal reef waters (28.5%). A diverse migratory life history of *M. miiuy* was observed based on Sr:Ba thresholds, and ultimately, we identified four migratory life histories of the species, including an estuary–coastal migratory type, a coastal resident type, a coastal–offshore migratory type and an estuary–coastal–offshore migratory type. This study provides a scientific basis for the protection of key habitats and seasonal management of *M. miiuy* in the ECS.

## 1. Introduction

Many coastal fish exhibit complex and diverse life histories and may traverse multiple habitats during their life cycle [1]. The variable biotic (e.g., prey availability) and abiotic (e.g., temperature, salinity) conditions of different habitats provide ontogenetic niches for feeding, reproduction, and overwintering [2]. The capacity for environmental migration improves the likelihood that any given individual will maximize survival and reproductive success, particularly in the face of stress or change [3]. Diversity of life history strategies is reflected not only in interspecific differences but also in intraspecific behavioral plasticity—variation between individuals of the same species [4,5,6]. Characterizing fish migratory life histories provides critical scientific insights into population connectivity, natal origins, and habitat requirements across different life stages, thereby deepening our understanding of species’ ecological strategies and spatial dynamics. For commercially valuable or ecologically significant species, these findings provide a scientific basis for resource recovery strategies, including the protection of natal origins and migratory corridors. [7,8].

Brown croaker, *Miichthys miiuy* (Basilewsky, 1855), is a warm temperate, demersal fish widely distributed in the northwestern Pacific which functions as a high trophic-level predator in coastal ecosystems [9]. This species migrates seasonally between estuary and waters further offshore. Studies suggest that the fish move from offshore to coastal waters in spring for feeding, shift to low-salinity estuary waters in summer and autumn for spawning, and then return to warmer waters to overwinter [10]. Salinity and temperature are considered the key environmental factors driving the seasonal migration of *M. miiuy* [11]. In recent years, populations of *M. miiuy* have declined due to high fishing intensity and environmental pollution [12]. There is an urgent need to clarify the migratory life history and habitat use of *M. miiuy* to underpin targeted strategies for resource recovery. However, studying the underwater movement and migration of individuals in the wild has always been a challenge, especially across broad spatial and temporal scales [13]. Traditional approaches for investigating fish migration have relied on mark-recapture, acoustic telemetry, and satellite tagging [14,15,16]. But these techniques are limited—tagging is difficult on smaller fishes, and even if tagged—tags have low likelihood of retention. Telemetry is also associated with high technological costs, meaning the approach may be unreliable to track total life history information—particularly if our interest includes younger age classes [17].

Otoliths are calcified structures in the inner ear of fishes that record environmental and physiological information throughout life. As natural archives, they are widely used for age determination, stock discrimination, diet analysis, and reconstruction of migratory histories [18,19,20,21]. Sequential analysis from the otolith core to the edge provides a record of the environmental characteristics experienced by the fish as it ages and is often used as a proxy for reconstructing fish origin and migratory pathways [22]. For example, the concentrations of strontium (Sr) and barium (Ba) in fish otoliths are primarily derived from the water, and the Sr:Calcium (Ca) and Ba:Ca ratios are used as proxies to estimate water salinity and distance from land [23,24,25,26]. In addition, the Sr:Ba ratio is particularly sensitive to transitions between estuarine and marine environments, making it an effective chemical tool for tracing fishes’ habitat shifts between these zones [15,27,28]. Interestingly, physiological processes also play a significant role in otolith growth and element incorporation [29]. For example, the Mg^2+^ content in otoliths has been found to correlate positively with fish metabolism. When combined with otolith microstructure features, the Mg^2+^ concentrations have been used to establish a “chemical clock” for age determination and life history stage classification [30]. Overall, otolith microchemistry provides a continuous record of fish life history across both temporal and spatial scales, offering unique advantages in revealing habitat-use patterns, migratory pathways, and their underlying drivers.

This study reconstructed the migratory life history of *M. miiuy* in the East China Sea (ECS) using otolith microchemistry, revealing habitat use and migratory characteristics across life stages and providing a scientific basis for resource management and conservation strategies. We sought, in this study, the following objectives:

(1) Develop the otolith magnesium chemical clock in combination with otolith microstructure as a temporal reference to support the reconstruction of migratory life history.
(2) Use typical tracer elements (Sr:Ca and Ba:Ca) in otolith cores to identify the water chemistry information of natal sources and establish Sr:Ba thresholds from estuary to offshore.
(3) Reconstruct the migratory life history of *M. miiuy* by combining information from the magnesium chemical clock and Sr:Ba of otoliths.

## 2. Materials and Methods

### 2.1. Fish Collection

**Figure 1:**
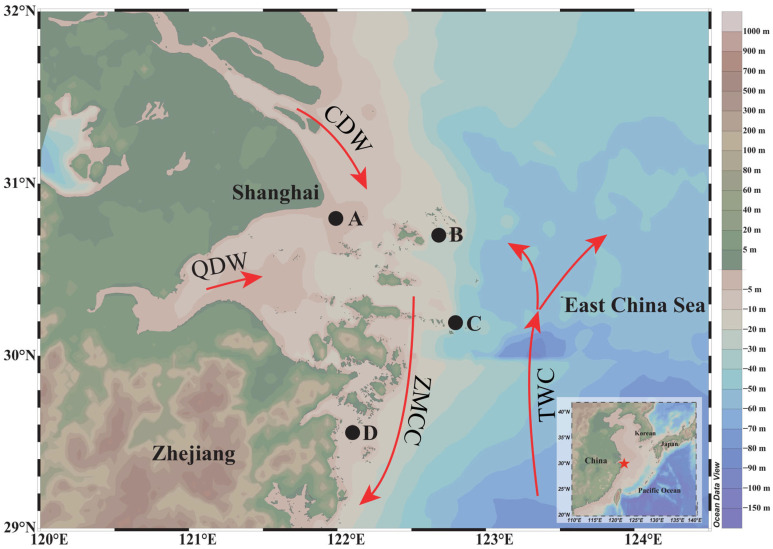
Sampling area of adult *M. miiuy* in the ECS. CDW: Changjiang Diluted Water; QDW: Qiantang Diluted Water; TWC: Taiwan Warm Current; ZMCC: Zhe–Min Coastal Current; Black dots indicate *M. miiuy* sampling locations. A (30.75° N, 122.00° E) and D (29.50° N, 122.12° E) were designated as estuarine sampling sites, while B (30.70° N, 122.74° E) and C (30.18° N, 122.80° E) were designated as coastal sampling sites.

Based on historical survey data from the Zhejiang Marine Fisheries Research Institute, spawning and feeding grounds of *M. miiuy* have been identified along the East China Sea coast. Therefore, sampling stations were established in four major fishing grounds for *M. miiuy* in this region (Figure 1), covering the main migratory corridors and habitats of *M. miiuy*. Samples were collected from May to September and were obtained from bottom-trawl fisheries and landings by local fishers. Immediately after fish were transported to shore, all specimens were frozen at −20 °C and transported to the laboratory for processing. After thawing, total length (TL, mm) and body weight (BW, g) were recorded, and both sex and gonadal maturity stage were determined during dissection (Table 1). Gonadal development and maturity were classified based on macroscopic morphological characteristics, including gonad size, color, vascularization, and the visibility of oocytes or milt. The staging followed the widely adopted criteria described by Ferreri et al. 2009, which categorize the gonads into six distinct developmental stages (Ⅰ–Ⅵ) [31]. Both sagittal otoliths were then carefully removed, cleaned of adhering tissues, rinsed with deionized water, and air-dried at room temperature. To ensure consistency in subsequent analyses, only the left sagittal otolith was used for microstructural observations and microchemical analyses. All samples in this study were collected through legal fishery operations. The experiments were approved by the Experimental Animal Care and Use Ethics Committee of Zhejiang Ocean University. Throughout the experimental procedures, all personnel strictly adhered to the ethical guidelines of Zhejiang Ocean University and complied with the institutional regulations by the university’s ethics committee.

### 2.2. Otolith Microchemistry Analysis

In this study, a total of 42 otolith samples were collected and embedded in epoxy resin (Epofix, Struers, Ballerup, Denmark) and sectioned along the transverse plane into slices (4–6 mm thick), using a precision low-speed saw (Buehler). The sections were ground sequentially with 500, 1000, 1200, and 2000 grit abrasive papers until the core was nearly exposed. Final polishing was performed using a polishing machine (RotoPol-35, Struers, Denmark) equipped with a woven cloth polishing pad and polishing suspension until the otolith core was fully exposed and the surface was free of visible scratches. The resin blocks containing the otoliths were ultrasonically cleaned in Milli-Q (MQ) water for 5 min and air-dried naturally. Following Xuan et al. 2023, the sequential change in elemental composition along the growth axis of the otoliths was analyzed using a laser ablation system (NWR213, New Wave Research, Fremont, CA, USA) coupled to an inductively coupled plasma mass spectrometer (Agilent 7500ce ICP-MS, Agilent Technologies, Santa Clara, CA, USA) [4]. The instrument settings were accelerating voltage 10 kV, wavelength 213 nm, pulse frequency 10 Hz, energy density 9.86 J/cm^2^, laser spot diameter 40 μm, scan speed 20 μm/s, and spacing of 10 μm between successive analytical points. NIST 612 and MACS-3 were used as reference standards and were re-analyzed after every 10 samples for calibration. A 100-s blank signal was collected before and after each analytical sequence and used as the background to calculate the limits of detection (LOD) for each element. Because trace element concentrations in otoliths are substantially lower than that of calcium, all elements were normalized to calcium following international convention and expressed as molar concentrations (X:Ca × 10^−3^ mmol mol^−1^). During the analysis, the relative standard deviation (RSD) of the corresponding elemental ratios in the standard signals was calculated to evaluate signal stability, and all results met the criterion of RSD% < 10 [4]. Based on the measurements of standard reference materials, the limits of detection (LOD, mmol mol^−1^) for each element relative to calcium were as follows: Mg:Ca (2.4 × 10^−3^), Sr:Ca (4.5 × 10^−5^), Ba:Ca (4.1 × 10^−5^).

### 2.3. Otolith Age Reading

Following the completion of microchemical analyses, the left sagittal otoliths were etched with 5% ethylenediaminetetraacetate (EDTA, pH 7.5, buffered with KOH) to enhance the visibility of growth annuli [32]. High-resolution otolith images were then captured using a Nikon SMZ800 (Nikon Corporation, Tokyo, Japan) reflected-light microscope equipped with a digital imaging system, and the distances from the otolith core to winter annuli were measured along the laser ablation transect to extract growth and microstructure information for subsequent age estimation. In this study, the age of *M. miiuy* was determined as the number of otolith winter annuli plus 1 year.

Age determination of *M. miiuy* was independently conducted by two experienced readers who were blinded to fish size and sampling date. The precision of age readings was initially evaluated based on independent counts by calculating the average percent error (APE) and coefficient of variation (CV) following Campana 2001 [33]. Generally, APE values below 5% and CV values below 10% are considered acceptable indicators of consistency between readers. In this study, the APE and CV between the two readers were 3.2% and 6.1%, respectively, demonstrating high precision and reproducibility of the age determinations. In cases where discrepancies occurred between the two readings, a joint re-examination was performed to reach a consensus, which was then used as the final age estimate. The calculation method of APE and CV is as follows:

(1) $$\mathrm{APE}=\frac{1}{N}\sum_{j=1}^{N}\left(\frac{\left|X_{\mathrm{ij}}-{\bar{X}}_{i}\right|}{{\bar{X}}_{i}}\times 100\right)$$

(2) $$\mathrm{CV}=\frac{1}{N}\sum_{i=1}^{N}\left(\frac{SD_{i}}{{\bar{X}}_{i}}\times 100\right)$$

where $X_{\mathrm{ij}}$ refers to the age assigned to the i*^th^* fish by the j*^th^* reader, ${\bar{X}}_{i}$ is the mean age estimate for the i*^th^* fish, ${SD}_{i}$ is the standard deviation of age estimates for that fish, N is the total number of fish examined.

### 2.4. Statistical Analysis

The Mg:Ca ratios obtained from sequential samples along the otolith growth axis were superimposed onto the corresponding otolith image, and the positions of the winter annuli where marked [34]. Along the scan line of laser ablation, the distance from the otolith core to the winter annulus (henceforth, winter annulus radius) (*A_i_*) and the distance from the first ablation spot to the location of the Mg:Ca troughs (henceforth, the chemical radius) (*B_i_*) were measured. A total of 42 otoliths were analyzed, yielding 82 paired measurements of Ai and Bi. Each measurement was repeated three times, and the mean value was used for subsequent analyses. Prior to conducting the Bland–Altman analysis, the normality of the 84 differences between Ai and Bi was assessed using the Shapiro–Wilk test, and the results confirmed that the data followed a normal distribution (*p* > 0.05). Bland–Altman analysis was used to assess test for a relationship between Ai and Bi. The calculation method of Limits of Agreement (*LoA*) is as follows [35]:

(3) LoA = bias ± 1.96 × SD

(4) $$\mathrm{bias}=\frac{1}{n}\;\sum_{i=1}^{n}\left(\;A_{i}-B_{i}\right)$$

(5) $$\mathrm{SD}=\sqrt{\frac{1}{n-1}\sum_{i=1}^{n}{\left(D_{i}-\mathrm{bias}\right)}^{2}}$$

where *LoA* refers to the range of agreement between two measurement methods. In this study, if the *LoA* range is less than (±1), it indicates that the two measurement results have a strong agreement. *Bias* is the average difference between the two measurement methods. 1.96 is a constant representing the 95% confidence interval. *SD* is the standard deviation of their differences. n is the number of paired measurements (number of individuals). *Ai* is the observation of i-th winter annulus radius in μm. *Bi* is the observation of i-th chemical radius in μm [36].

The edge of the otolith has grown most recently and consequently represents the most recent environment preceding capture [22]. The ‘otolith edge’ was defined as the final four points (i.e., 30 µm) along the scan line [37]. To investigate seasonal variation of the Mg:Ca ratio, we collected 42 samples across multiple months and compared average monthly edge Mg:Ca means across samples using a Kruskal–Wallis test. When significant differences were detected, pairwise comparisons were conducted with Dunn’s multiple comparison test to identify which specific months are significantly different from others (Statistical significance was accepted at *p* < 0.05). To visualize monthly Mg:Ca trends, we applied LOESS smoothing to the monthly means with 95% confidence bands.

The otolith core was defined as the first four points (i.e., 30 µm) along the scan line [37]. The elemental composition of the core is thought to reflect the oldest tissue and is consequently used as a proxy for estimating the hydrochemical characteristics of a fish’s natal habitat [22]. To investigate whether distinct natal sources of *M. miiuy* could be distinguished, we performed K-means clustering on the mean Sr:Ca and Ba:Ca ratios measured from 42 otoliths cores, employing Euclidean distance as the dissimilarity metric and determining the optimal number of clusters (*K*) based on the elbow criterion. Prior to clustering, data normality was examined using the Shapiro–Wilk test, which indicated that the variables did not conform to a normal distribution (*p* < 0.05). A minimum cluster size of five individuals was specified to avoid the formation of spurious small clusters [38]. Then, we tested for differences in the mean Sr:Ba values of the core among groups (Statistical significance was accepted at *p* ≤ 0.05). Homogeneity of variance was assessed using Levene’s test, and normality was examined with the Shapiro–Wilk test. When data met both assumptions, independent-samples *t*-tests were used; otherwise, nonparametric Mann–Whitney tests were used, with Bonferroni correction for multiple comparisons. Based on the group differences and distributions of Sr:Ba values, we established Sr:Ba thresholds for estuary to coastal environments. Given that Sr:Ba ratios increase with distance offshore, observations with Sr:Ba exceeding the upper limit of natal-source thresholds were provisionally interpreted as indicative of offshore signals [27,39,40]. To verify the applicability of the established Sr:Ba thresholds, we used the mean Sr:Ba of otolith edge as the chemical signature representing the capture location of *M. miiuy* and applied it for threshold validation.

A bivariate time series developed to detect stationary and transitional phases in multidimensional time series, such as animal movement trajectories, has recently been applied to otolith microchemistry to segment elemental ratio profiles and identify fish habitat transitions [38]. In order to trace the migratory life history of *M. miiuy*, bivariate time series based on Mg:Ca and Sr:Ba ratio were screened to identify change points and then partitioned to produce segments [41]. Each segment is assumed to represent sedentary phases in distinct habitats. The Mg:Ca value is used to indicate the life stage, and the Sr:Ba value is used to indicate the migration behavior. The Bayesian Information Criterion (BIC) was employed to determine the optimal number of segments, and the model with the lowest BIC value was selected as the final solution. A minimum segment length of four measurements (i.e., length of 30 μm on the otolith) has been specified for the segmentation process to avoid over-segmentation [42]. Data processing and analysis in this study were performed in Excel 2021, SPSS 22.0, and R 4.5.0.

## 3. Results

### 3.1. Otolith Mg:Ca Chemical Clock

**Figure 2:**
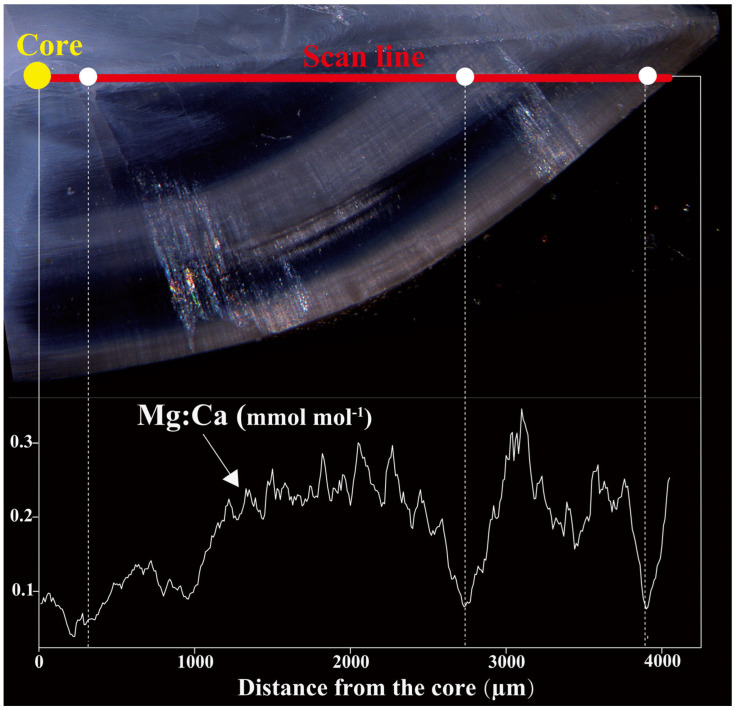
The Mg:Ca transect in the otolith of *M. miiuy* from the ECS is overlaid on the corresponding otolith image, showing the spatial distribution of Mg:Ca in relation to optical features. Lowest concentrations are interpreted as the positions of winter annuli and are marked with white dots and vertical dashed lines.

**Figure 3:**
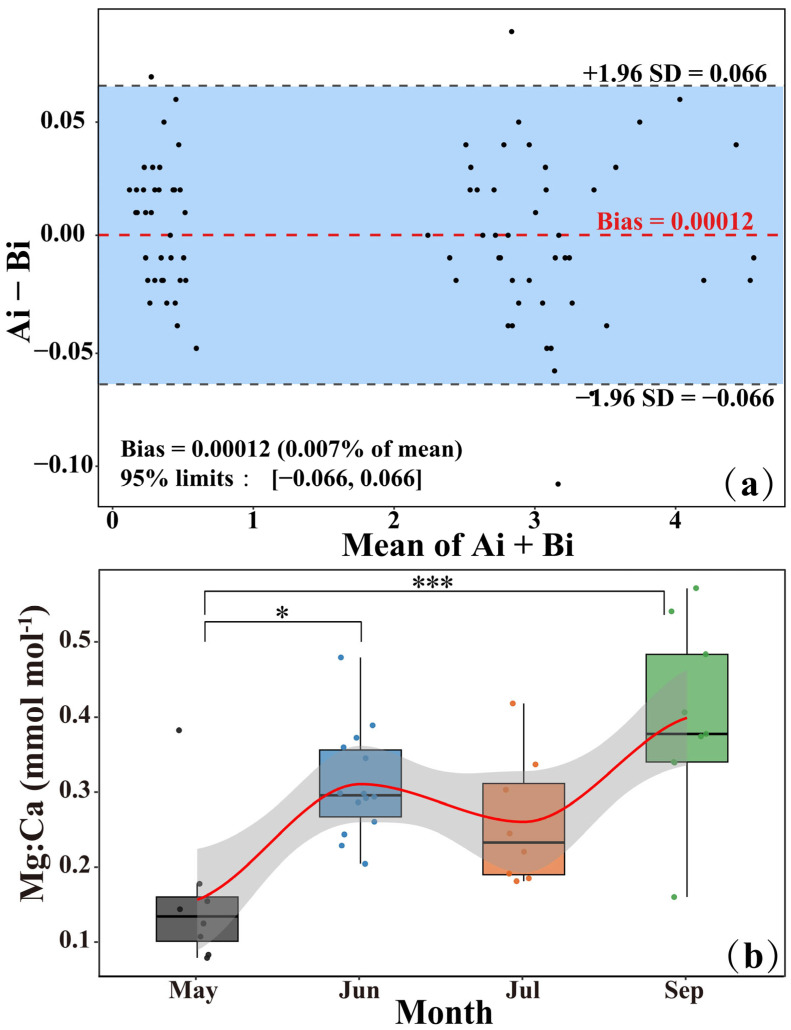
(**a**) Bland–Altman plot comparing the positional alignment between winter annulus radius (*Ai*) and chemical radius (*Bi*); (**b**) Seasonal variation in edge Mg:Ca ratios. Box plots show medians, interquartile ranges, whiskers extend to the most extreme values within 1.5 × IQR. Asterisks indicate significant differences among months (* *p* < 0.05; *** *p* < 0.001). The red line with shaded area shows Loess smoothing and its 95% confidence interval.

The otolith Mg:Ca ratio exhibited a sinusoidal pattern with alternating peaks and troughs along the scan line. Troughs coincided with opaque zones, whereas peaks aligned with translucent zones (Figure 2). Winter annulus radius (*Ai*) and chemical radius (*Bi*) showed minimal deviation (mean difference = 1.2 × 10^−4^) with narrow 95% *LoA* (−0.066 to 0.066), indicating that the measurements are very consistent with each other (Figure 3a). This confirms a robust positional match between Mg:Ca troughs and annual winter annuli. Furthermore, the mean Mg:Ca ratio at otolith edge varied significantly among months (*p* ≤ 0.05), with significant differences between May and June (*p* ≤ 0.05), and highly significant differences between May and September (*p* ≤ 0.001), while June and July did not differ significantly (*p* > 0.05). LOESS smoothing produced a wavelike trend in edge Mg:Ca, with values rising from May to September, reaching a peak near September (Figure 3b).

### 3.2. Natal Sources and Migratory Life History

**Figure 4:**
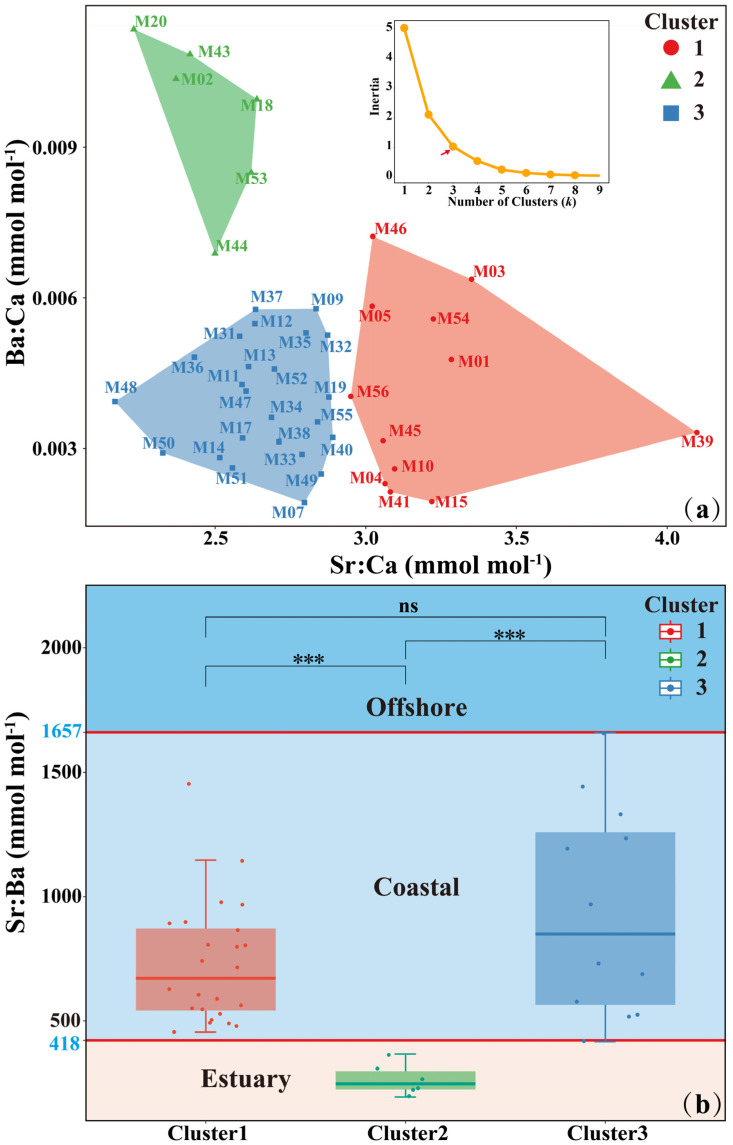
(**a**) K-means clustering results with the optimal number of clusters (The red arrow in the elbow plot indicates the optimal number of clusters (*K* = 3), at which the within-cluster sum of squares exhibits a clear inflection point.) based on otolith Sr:Ca and Ba:Ca. Each colored polygon represents the convex hull of individuals within a cluster. (**b**) Boxplots of otolith Sr:Ba values for three clusters, with overlaid jittered points showing individual observations. Boxes indicate interquartile ranges (IQR), horizontal lines represent medians, and whiskers extend to the most extreme values within 1.5 × IQR. Asterisks indicate significant differences among months (ns *p* > 0.05; *** *p* < 0.001). Each whisker terminates at the furthest observation within this range, while points beyond whiskers represent outliers. The red horizontal line indicates the Sr:Ba threshold, while different background colors delineate the estuarine, coastal, and offshore waters.

Cluster analysis suggested three groups with clear differences in elemental concentrations (Figure 4a). Cluster 1, characterized by the highest Sr:Ca values (3.02~4.09 mmol/mol) and relatively low Ba:Ca values (0.0019~0.0072 mmol/mol), accounted for 28.5% of individuals; Cluster 2, with lower Sr:Ca values (2.22~2.63 mmol/mol) and the highest Ba:Ca values (0.0068~0.0113 mmol/mol), accounted for 14.2% of individuals; Cluster 3, characterized by lower Sr:Ca values (2.16~2.89 mmol/mol) and lower Ba:Ca values (0.0019~0.0113 mmol/mol), accounted for 57.3% of individuals.

Shapiro–Wilk tests indicated that Sr:Ba values from Cluster 1 deviated from normality (*p* ≤ 0.05), whereas Cluster 2 (*p* > 0.05) and Cluster 3 (*p* > 0.05) satisfied the normality assumption. Levene’s test indicated heteroscedasticity among the three groups (*p* ≤ 0.05). Kruskal–Wallis H test suggested significant differences in Sr:Ba among the three clusters (H = 16.21, *p* ≤ 0.05). Pairwise Mann–Whitney U tests with Bonferroni correction revealed significant differences between Cluster 1 and Cluster 2 (*p* ≤ 0.05) and between Cluster 2 and Cluster 3 (*p* ≤ 0.05), but no significant difference between Cluster 1 and Cluster 3 (*p* > 0.05) (Figure 4b). Accordingly, in this study, the Sr:Ba value range represented by Clusters 1 and 3 was defined as the chemical signature of coastal habitat; values lower than this range were interpreted as estuary habitat, and values higher than this range were interpreted as offshore habitat (Figure 4b). Using mean Sr:Ba ratios (541~1820 mmol mol^−1^) of otolith edge from different sampling locations, we validated the threshold with 92.9% accuracy.

**Figure 5:**
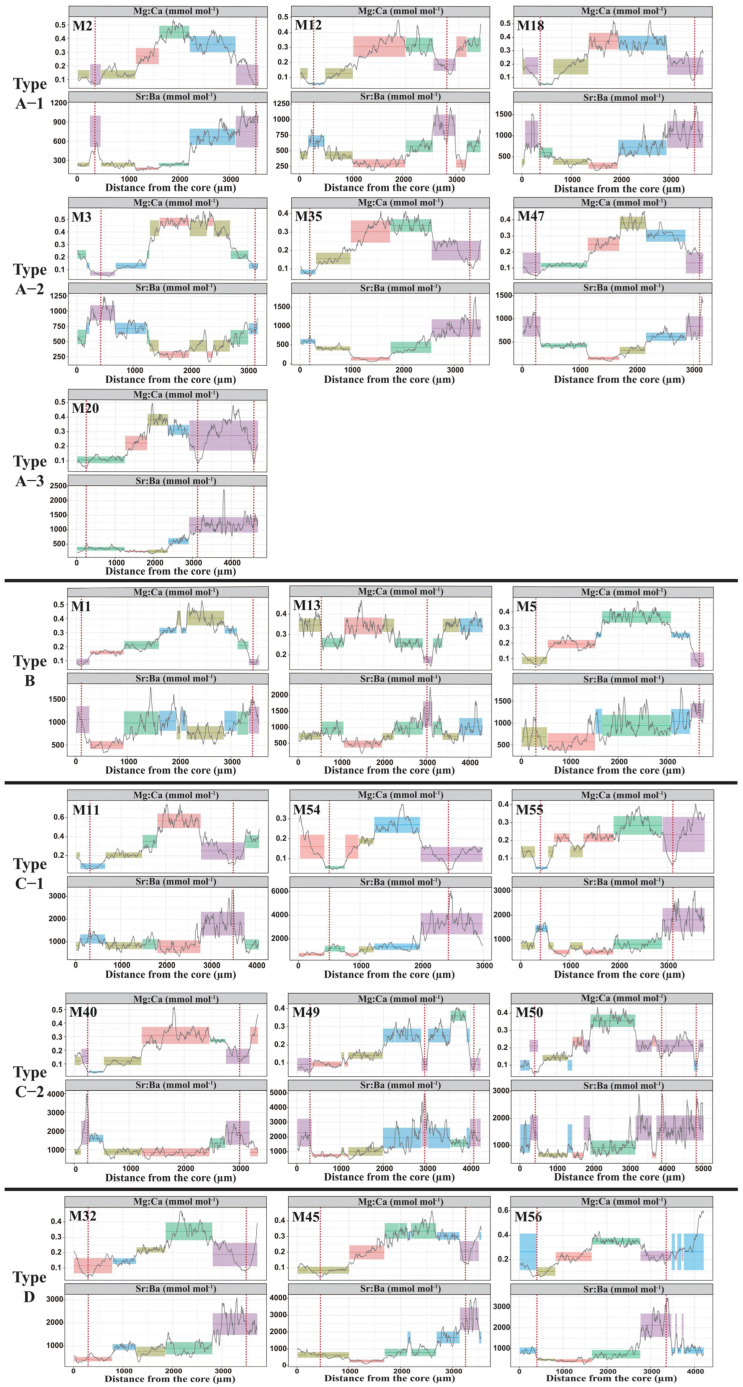
Otolith transects of *M. miiuy* showing Mg:Ca (upper panels) and Sr:Ba (lower panels) ratios plotted against distance from the core. Mg:Ca is used as a temporal indicator, while Sr:Ba reflects habitat use. Colored background shading and line segments denote mean values of Mg:Ca and Sr:Ba for different segments, and red dashed lines indicate winter annuli. Based on the water environments experienced during the life cycle, four migratory life history types were identified: Type A (estuary–coastal migrants), Type B (coastal residents), Type C (coastal–offshore migrants), and Type D (estuary–coastal–offshore migrants). A maximum of three individuals will be displayed for each type. MX (X = numbers) denotes different sample numbers of *M. miiuy*.

**Figure 6:**
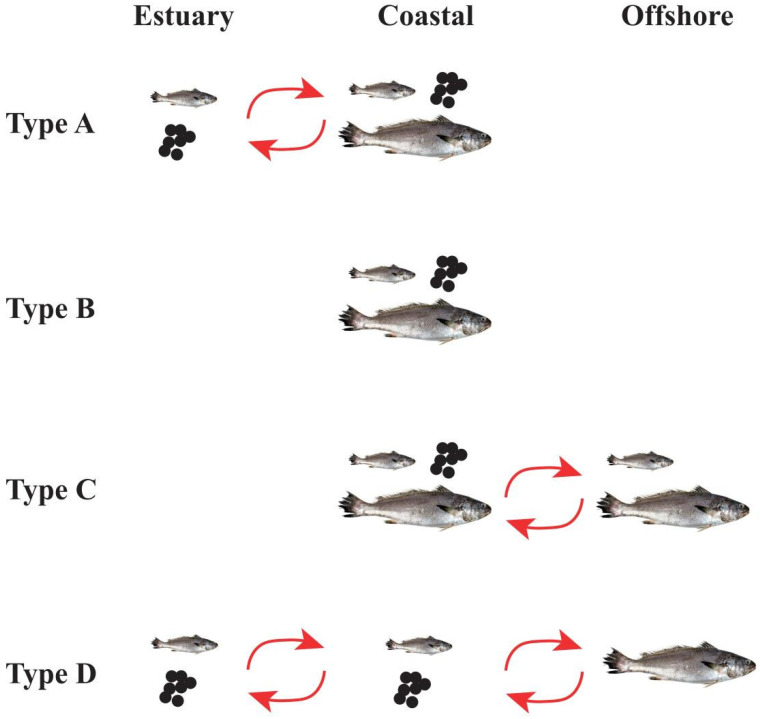
Four migratory life histories recognized for *M. miiiuy*. The black small circles represented fish eggs, and the sizes of the fish from small to large represented the juvenile, and adult stages, respectively. Type A: estuary–coastal migrants; Type B: coastal residents; Type C: coastal–offshore migrants; Type D: estuary–coastal–offshore migrants.

Results from the bivariate time series show that, for most individuals, Mg:Ca and Ba:Ca exhibit opposite trends and the Ba:Ca value typically peaked during the winter annulus. However, the Ba:Ca value range did vary among individuals (Figure 5). According the Sr:Ba thresholds of different habitats, we found that *M. miiuy* is widely distributed in estuaries, coastal and offshore waters, and identified four types of migration life history of *M. miiuy* based on their migratory behavior in these three habitats (Figure 6): Estuary–coastal type (Type A): Individuals of this type were characterized by mean Sr:Ba values of all segments being less than 1657, with some segments below 418; Coastal resident type (Type B): Individuals of this type had mean Sr:Ba values of all segments within 418~1657, with obvious increases near the winter annulus; Coastal–offshore type (Type C): Individuals of this type had mean Sr:Ba values of all segments distributed both within 418~1657 and above 1657; Estuary–coastal–offshore type (Type D): Individuals of this type exhibited mean Sr:Ba values spanning all three habitat ranges (below 418, 418~1657, and above 1657).

## 4. Discussion

### 4.1. Feasibility of Using Mg:Ca as a Chemical Clock for M. miiuy

The ECS is located in a subtropical monsoon climate zone with distinct seasonal temperature differences; water temperature is lowest in winter and highest in summer [10]. The seasonal variation of Mg:Ca ratios observed in this study is highly consistent with the seasonal changes in water temperature: Mg:Ca values in otoliths peak during high-temperature periods (summer), and decreased significantly during low-temperature periods (winter). This result is consistent with the findings of Limburg et al., 2018, on *Atlantic cod* and *Baltic herring* [30]. However, whether water temperature is the main factor driving the periodic variation of Mg:Ca ratios in *M. miiuy* otoliths remains controversial. Numerous studies have shown that the Mg^2+^ deposition in otoliths is not directly linearly related to temperature but is mainly regulated by physiological processes such as growth and metabolism [43,44,45]. These processes, while influenced by temperature, are also tightly linked to feeding behavior, reproductive activity, energy allocation, and metabolic rates, with complex interactions among them—for example, elevated temperatures can stimulate feeding intensity and increase metabolic demand [46]. In *Reinhardtius hippoglossoides*, which inhabits a non-seasonal, deep-cold environment, Mg:Ca distribution patterns are more strongly associated with individual physiological conditions and early growth history than with external environmental cycles. In contrast, the *Platichthys flesus* inhabiting highly seasonal environments show clear periodic Mg:Ca fluctuations that align with growth rhythms and seasonal activity patterns [47,48]. Together, these findings indicate that Mg:Ca reflects a complex integration of environmental influences and internal physiological states rather than a simple temperature signal [30,34]. We found no significant difference in Mg:Ca ratios among individuals at different gonadal development stages (*p* > 0.05); even in age stages 0–2, which had not yet reproduced, distinct seasonal fluctuations in Mg:Ca ratios were observed. This indicates that reproductive activity is not the dominant factor causing this seasonal variation. Previous studies have proposed a potential two-step mechanism: magnesium first enters the otolith endolymph in a limited manner through ion channels and subsequently binds with water-soluble proteins within the chamber [30]. This process likely links Mg^2+^ incorporation to cellular-level regulation and metabolic activity.

In summary, although the specific physiological regulatory mechanisms controlling Mg^2+^ deposition remain unclear, the results of this study provide empirical support for the concept of an “otolith chemical clock,” indicating that Mg:Ca values can serve as an effective chemical indicator to estimate age and life history stage of *M. miiuy* in the coastal waters of the ECS. Building upon this temporal foundation, the subsequent analysis integrates Mg-derived chronological information with other elemental markers (e.g., Sr:Ca and Ba:Ca) to infer the natal sources and migratory history of *M. miiuy*.

### 4.2. Natal Sources

Ba:Ca ratios are negatively correlated with salinity, whereas Sr:Ca ratios are positively correlated [49]. Consistent with this, marine fish that hatch or reside in estuarine waters typically exhibit low Sr:Ca and high Ba:Ca signatures [4]. In this study, Cluster 2 individuals exhibited higher Ba:Ca and lower Sr:Ca values in their otolith cores, suggesting an estuarine origin. This inference is supported by regional hydrological features: the Yangtze River, China’s largest river in terms of discharge, delivers massive freshwater input, reducing salinity in the estuary and adjacent waters while increasing the concentration of dissolved trace elements (particularly metals) [50]. Moreover, the area is strongly influenced by coastal upwelling, which transports Ba-enriched bottom water to the surface, further elevating Ba concentrations [51,52]. Based on these hydrogeochemical characteristics, it is likely that the natal estuaries for Cluster 2 are waters adjacent to the Yangtze Estuary. This interpretation is consistent with otolith microchemical studies of both *Larimichthys polyactis* and *Eleutheronema tetradactylum* which each have significantly higher Ba:Ca values in otolith samples from the Yangtze Estuary compared to other coastal regions such as the southern Yellow Sea, the ECS, and the South China Sea [4,53]. Similarly, higher Ba:Ca values have been shown to effectively identify *Larimichthys polyactis* originating from the Yangtze Estuary [54]. Clusters 1 and 3 both exhibited lower Ba:Ca ratios than Cluster 2, suggesting they were not originally from estuarine waters. Cluster 3, however, had Sr:Ca ratios significantly lower than Cluster 1 and slightly higher than Cluster 2. To date, there is no evidence indicating that *M. miiuy* spawns in oceanic waters. Accordingly, Cluster 3 likely represents a coastal mixed-water spawning ground, whereas Cluster 1 may correspond to a coastal reef-water spawning ground where far from major estuaries and less affected by freshwater input, such as the Zhoushan Archipelago or the southern Yellow Sea. Notably, one individual in Cluster 3 (M39) exhibited an otolith core Sr:Ca value (4.09 mmol/mol) much higher than all other individuals, closely matching the levels observed in *M. miiuy* samples from the southwest Yellow Sea [55]. This suggests that M39 may have originated from that region.

Our results demonstrate that over 14% of *M. miiuy* individuals originated from the estuary and more than 57% from the coastal mixed waters. Estuarine ecosystems provide sufficient food sources and favorable conditions for eggs and larvae [56]. However, estuaries also pose high predation risks and intense inter- and intraspecific competition. Thus, some fishes adopt a “peripheral spawning” strategy, spawning in coastal mixed waters to balance the benefits of high nutrient availability with reduced ecological risk [57] *M. miiuy*’s preference for coastal mixed waters may reflect this ecological trade-off. This distribution pattern is consistent with studies of other Sciaenidae species, such as *Larimichthys polyactis*, *Collichthys lucidus*, and *Nibea albiflora*, which all establish spawning and nursery grounds in waters surrounding estuaries [53,58,59]. We did observe, however, that more than 20% of *M. miiuy* originate from coastal reef waters, which may be an ecological or evolutionary decision designed to reduce intraspecific competition and human disturbance [60]. The capacity of the species to choose from diverse natal sources helps distribute risk from the degradation of any single one, enhance adaptability to environmental stress, and provide an “ecological insurance effect” [61]. Moreover, because some spawning grounds may share similar elemental signatures, the existence of additional natal sources cannot be entirely ruled out.

### 4.3. Diversity of Migratory Life History

In this study, all specimens were collected from the ECS and were primarily age-3 (with some age-4) that had completed a full life history cycle, including reproduction and overwintering. Although sampling was conducted mainly during the warm season (May–September), this does not bias the interpretation of annual migratory patterns, as the incorporation of Sr^2+^ and Ba^2+^ into otoliths is primarily controlled by ambient concentrations rather than temperature effects [62]. Bivariate time series analysis showed that 84% *M. miiuy* were migratory, whereas 16% were coastal residents that did not migrate across habitats. Below, we discuss the four migratory life histories identified in our analysis (Figure 5 and Figure 6).

Estuary–coastal type (Type A): refers to individuals living in estuary and coastal habitats. Among them, three individuals (Type A-1) that hatched in the estuary quickly migrated to coastal waters after a short stay. *M. miiuy* has a spawning peak from September to October [10], meaning that juveniles face their first winter shortly after hatching. In winter, the temperature of estuary waters is lower than that of coastal areas [63]. Therefore, once juveniles acquire sufficient swimming ability, they rapidly leave the estuary and migrate to warmer coastal environments. In contrast, one individual remained in the estuary for a prolonged period and did not migrate to coastal waters until the following winter (Type A-3). This one may have delayed development or weak swimming ability, lacking the capacity to migrate earlier. Previous studies have reported that in species such as *Salvelinus malma*, slower-growing individuals often delay marine migration [64]. Notably, most individuals of type A (10 individuals) hatched in coastal waters, remained there throughout the first winter, then migrated to the estuary following spring to feed (Type A-2). During their feeding period, otolith Sr:Ba ratios fluctuated between estuarine and coastal thresholds, suggesting that the transition zones are important feeding grounds for the *M. miiuy* population. Unpublished gut-content analyses of juvenile *M. miiuy* from our group indicate that small- to medium-sized shrimp constitute the primary prey during the juvenile stage; in the ECS, these shrimps are most abundant in estuary–coastal transition waters [65].

Coastal resident type (Type B): Individuals of this type lived in coastal waters throughout their life cycle, with no significant habitat transitions. This life history characteristic is consistent with some *Larimichthys crocea* in this area [66]. The coastal waters of the East China Sea (ECS) offer distinct ecological advantages, including numerous islands and reefs, varied benthic substrates, and seasonally favorable temperatures, that collectively provide suitable conditions for the reproduction, growth, and overwintering of *M. miiuy* [67]. Consequently, some *M. miiuy* do not need to undertake long-distance migrations and instead complete their entire life cycle within this coastal region.

Coastal–offshore type (Type C): Most individuals of this type (n = 12) resided in coastal waters throughout their first year of life, with no migratory behavior observed. After that, they moved to offshore waters for overwintering in their second winter. After overwintering, some individuals returned to coastal waters, while others remained offshore (Type C-1) [64]. In addition, a few individuals migrated to offshore waters for overwintering during their first winter (Type C-2). This indicates that *M. miiuy* already possess the ability to inhabit offshore waters during the juvenile stage. This contrasts with other, similar species, such as *Argyrosomus hololepidotus* that migrate offshore only after reaching adult size. [26]. Then these *M. miiuy* travel back and forth between coastal and offshore.

Estuary–coastal–offshore type (Type D): Individuals of this type traveled back and forth from estuary to offshore waters. For example, individual M32 hatched in the estuary and overwintered there, migrated to coastal habitats the following spring for feeding, and subsequently moved to a marine environment for a second overwintering. In contrast, individuals M45 and M56 hatched in coastal habitats and completed their first overwintering there before migrating to the estuary the following spring for feeding and then moving through coastal waters to offshore habitats for overwintering. The east–west long-distance migration displayed by *M. miiuy* differs from the north–south continental shelf migration observed in *Eleutheronema tetradactylum* [28]. This difference indicates that *M. miiuy* possesses higher salinity tolerance and greater ecological plasticity [68].

## 5. Conclusions

Otolith natural markers provided an effective method for studying the natal sources and migratory life history of *M*. *miiuy* in the East China Sea. Our results demonstrated that most individuals originating from estuary and coastal waters exhibited seasonal migrations, flexibly moving among estuary, coastal, and offshore waters. In addition, a small proportion of individuals with coastal natal sources did not perform cross-habitat migrations but instead remained resident in coastal waters throughout their lives. These findings supplemented our understanding of the migratory history of *M. miiuy* and provided important insights for the conservation of critical estuary–coastal habitats and the sustainable management of its fisheries. We suggest that implementing seasonal fishing bans in estuarine and coastal waters during the spawning season could help protect spawning adults. Protecting key estuarine and coastal nursery grounds and restricting intensive fishing in these areas would improve juvenile survival rates. Furthermore, establishing ecological corridors and maintaining habitat connectivity would ensure the smooth completion of migratory behaviors, ultimately supporting the long-term recovery and sustainable exploitation of *M. miiuy* populations.

## Figures and Tables

**Table 1:** Monthly sample sizes and biological characteristics of *M. miiuy*.

Month	Numbers	SL (mm, Mean ± SD)	BW (g, Mean ± SD)	Sex Ratio (♀:♂)	Stage of Sexual Maturity
May	8	370.5 ± 38.1	423.8 ± 140.2	3:1	Ⅰ–Ⅱ
Jun	17	451.3 ± 90.6	997.4 ± 546.0	7:1	Ⅱ
Jul	8	517.8 ± 139.2	3228.4 ± 1626.5	7:1	Ⅱ–Ⅲ
Sep	9	541.2 ± 37.0	1314.5 ± 347.6	4:3	Ⅱ–Ⅵ

Note: SL: Standard length; BW: Body weight. The stage of sexual maturity was determined macroscopically.

## Data Availability

The original contributions presented in the study are included in the article. Further inquiries can be directed to the corresponding author.
